# Supplementary material for: A Multi-Domain Group-Based Intervention to Promote Physical Activity, Healthy Nutrition, and Psychological Wellbeing in Older People with Losses in Intrinsic Capacity: AMICOPE Development Study
Source: Int J Environ Res Public Health. 2021 Jun 2;18(11):5979. doi: 10.3390/ijerph18115979 (PMC8199683; doi:10.3390/ijerph18115979)
Supplement: Supplementary file 1 [file ijerph-18-05979-s001.zip › ijerph-1238026-supplementary.pdf]

The TIDieR (Template for Intervention Description and Replication) Checklist			
**Item number**	**Item**	**Where located in primary paper (page)**	**Manuscript**
1.	**BRIEF NAME.** Provide the name or a phrase that describes the intervention.	6	“The intervention is called “AMICOPE” (Aptitude Multi-domain intervention to promote Intrinsic Capacity in Older PEople)”
2.	**WHY.** Describe any rationale, theory, or goal of the elements essential to the intervention.	6	“It is aimed at promoting physical activity, healthy nutrition and psychological wellbeing in older people living in the community. The guiding principle of our work was the ICOPE strategy and the conceptual framework of the intervention is described in fig. 2.”
3.	**WHAT.** Materials: Describe any physical or informational materials used in the intervention, including those provided to participants or used in intervention delivery or in training of intervention providers. Provide information on where the materials can be accessed (e.g. online appendix, URL	7	The materials to be used in the intervention consist in a detailed guide for the facilitators (Version 1.0, ©Fundació Salut i Envelliment UAB) that will be available after refinement under a Creative Commons 4.0 License, CC BY., and additional resources such as maps, photographs, and audio files with free intellectual property rights used to perform some other activities.
4.	**WHAT.** Procedures: Describe each of the procedures, activities, and/or processes used in the intervention, including any enabling or support activities.	7	The intervention procedures for physical activity will be based on the Vivifrail© multi-component training program (©Mikel Izquierdo).<br>Several group dynamics will be performed to promote social support and exchange of personal experiences among participants, as well as the acquisition of self-management skills. Goal setting will be used to promote behavioural changes in the daily life of participants that are meaningful for them and that positively affect their healthy nutrition and psychological wellbeing.
5.	**WHO PROVIDED.** For each category of intervention provider (e.g. psychologist,	7	A pair of health and social care professionals with different background (nurse, physiotherapist, occupational

	nursing assistant), describe their expertise, background and any specific training given.		therapist, nutritionist, psychologist, physical activity trainer, etc.) will be previously trained as group facilitators by the research team, during a 30-hour training program based on how to apply the intervention guide.
6.	**HOW.** Describe the modes of delivery (e.g. face-to-face or by some other mechanism, such as internet or telephone) of the intervention and whether it was provided individually or in a group.	7	The intervention will consist of 12 face-to-face sessions facilitated in groups of 8 to-12 frail older people.
7.	**WHERE.** Describe the type(s) of location(s) where the intervention occurred, including any necessary infrastructure or relevant features.	7, 8	The intervention will be delivered in community facilities such as senior leisure centers, civic centers, or primary care centers, and in different locations of the surroundings. Particularly, 10 of the 12 sessions will take place in a space large enough to do physical activity. For one session the whole group will move to a grocery store to learn about nutritional facts. The remaining session will be devoted to visit another senior center with the purpose to know about programs and activities addressed to the community.
8.	**WHEN AND HOW MUCH.** Describe the number of times the intervention was delivered and over what period of time including the number of sessions, their schedule, and their duration, intensity or dose.	7	Sessions will be held weekly for 2.5 hours during three months. Each session will include one hour of physical exercise using the Vivifrail© program, and 1.5 hours dedicated to any other intervention components.
9.	**TAILORING.** If the intervention was planned to be personalised, titrated or adapted, then describe what, why, when, and how.	8	The physical activity domain of AMICOPE includes individual prescription passports for participants tailored to their individual functional capacity, which will be assessed by the Short Physical Performance Battery, a walking speed test, and the risk of falls.
10.	**MODIFICATIONS.** If the intervention was modified during the course of the study,	6	Due to the iterative and complex nature of the intervention development process, some aspects were discussed and some changes affected the scope of the intervention.

	describe the changes (what, why, when, and how).		Hence, and even though it was beyond the intervention's initial aims, specific contents about cognitive stimulation and medication review were suggested by the working group and finally incorporated as part of the final resulting intervention.
11.	**HOW WELL.** Planned: If intervention adherence or fidelity was assessed, describe how and by whom, and if any strategies were used to maintain or improve fidelity, describe them.	8	The intervention will be monitored by facilitators (or external observers during the pilot) with quantitative and qualitative indicators of adherence.
